# Phylogenetic Profiling of the Diabetic Foot Ulcer Microbiome of an Afro‐Caribbean Population

**DOI:** 10.1002/mbo3.70329

**Published:** 2026-06-10

**Authors:** Nkemcho Ojeh, Bidyut R. Mohapatra, Margaret O'Shea, Dale Springer, Judy Ward, Mohmmed Sallu, Natacha Paquette, Keith Gooding, Anna Springer, O. Peter Adams

**Affiliations:** ^1^ Department of Preclinical and Health Sciences, Faculty of Medical Sciences The University of the West Indies Bridgetown Barbados; ^2^ Department of Biological and Chemical Sciences The University of the West Indies Bridgetown Barbados; ^3^ Department of Clinical Sciences, Faculty of Medical Sciences The University of the West Indies Bridgetown Barbados

**Keywords:** diabetic foot ulcer, fungi, microbial communities, microbiome, next‐generation sequencing, wound healing

## Abstract

Diabetic foot ulcers (DFUs) are associated with high morbidity, amputation rates, and healthcare costs in Barbados. This pilot study compares the microbiome diversity of chronic DFUs and paired normal skin (controls) from biopsies in Afro‐Caribbean patients with type 2 diabetes using Illumina amplicon sequencing targeting the 16S ribosomal RNA (rRNA) gene and the internal transcribed spacer 2 (ITS2) region. Both DFUs and controls harbored diverse bacterial and fungal communities, with differences in taxonomic composition and relative abundance profiles. The dominant bacterial genera were *Corynebacterium* (18.3% in DFUs, 24.3% in controls) and *Staphylococcus* (14.9% in DFUs, 14.1% in controls). The dominant bacterial species was *Corynebacterium striatum* (17.3% in DFUs, 23.8% in controls) followed by *Pseudomonas aeruginosa* in DFUs (8.9%) and *Staphylococcus aureus* in controls (13.3%). The dominant fungal genera was *Densospora* (12% in DFUs, 12.6% in controls). The dominant fungal species was *Rhodotorula graminis* in DFUs (6.18%) and *Paracamarosporium leucadendri* in controls (3.85%). *Candida duobushaemulonii*, with intrinsic resistance to antifungal agents, was detected with higher relative abundance in DFUs than in controls (4.44% vs. 2.36%). Fungal Shannon alpha diversity was significantly reduced in DFUs (*p* = 0.039), while beta diversity did not differ between groups for bacteria (*p* = 0.982) or fungi (*p* = 0.975). The differences in taxonomic composition and relative abundance profiles, and co‐occurrence of clinically relevant bacterial and fungal taxa, highlight the potential role of polymicrobial communities in DFU chronicity in the Afro‐Caribbean cohort studied, and supports future studies to evaluate implications for antimicrobial stewardship.

## Graphical abstract

Illumina amplicon sequencing revealed diverse bacterial and fungal communities in chronic diabetic foot ulcers (DFUs) and adjacent normal skin from Afro‐Caribbean patients. *Corynebacterium striatum* dominated both sites. DFUs had higher relative abundance of *Pseudomonas aeruginosa* and the antifungal‐resistant *Candida duobushaemulonii*, with significantly reduced fungal Shannon alpha diversity (*p* = 0.039).

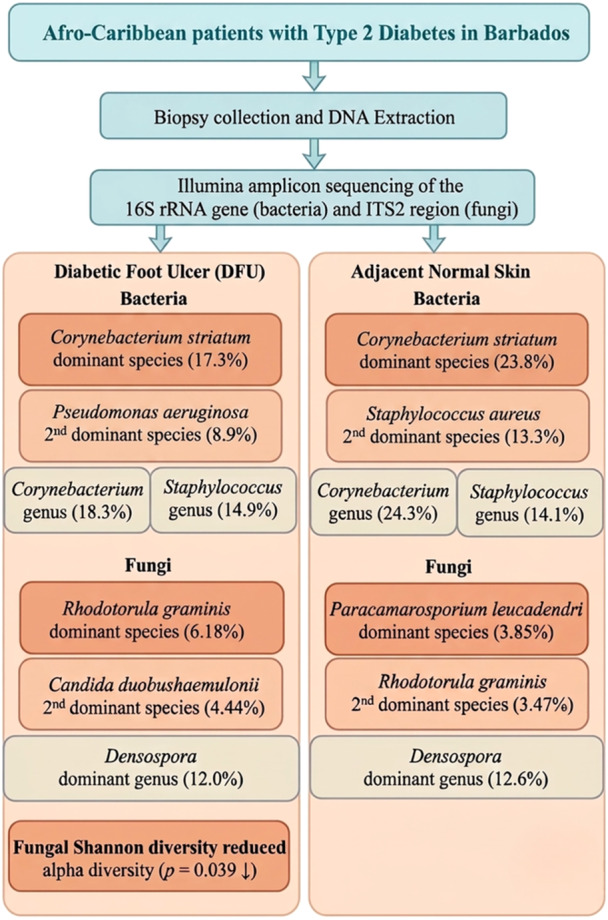

## 1 Introduction

Diabetic foot ulcers (DFUs) are common with the lifetime risk of a person with diabetes developing one estimated to be 19%–34% (McDermott et al. 2023). DFUs may severely impact quality of life because they are often chronic, painful and predispose to serious sequelae such as soft tissue infection, sepsis, osteomyelitis (Rodríguez‐Rodríguez et al. 2022) and precede around 80% of diabetes‐related lower extremity amputations (Lovell et al. 2023).

Barbados, a small‐island Eastern Caribbean state of approximately 280,000 people, 92% of African origin, has an estimated DFU prevalence of 14.7% (Lovell et al. 2023). Peripheral neuropathy and peripheral arterial disease predispose to DFUs and both have a high prevalence in people with diabetes in Barbados (Adams et al. 2019, 2022). The island has an estimated 936 per 100,000 diabetes‐related lower limb amputation rate (Hennis et al. 2004). There is a significant burden to the healthcare system with DFUs accounting for 89% of all diabetes‐related hospital admissions in Barbados (Taylor et al. 2014) and having an estimated annual direct and indirect healthcare services costs of US$12.2 M and US$4.1 M, respectively (Greenidge et al. 2022a, 2022b).

DFUs are non‐healing wounds that fail to progress through the normal wound healing stages in a timely manner (Nunan et al. 2014). The pathophysiology of DFU is multifactorial and includes infection, neuropathy, fibrosis, compromised immune status, ischemia and vasculopathy, all of which contribute to impaired healing (Eming et al. 2014; Tomic‐Canic et al. 2020). They exhibit a hyperproliferative and non‐migratory epidermis, along with dysregulated and prolonged inflammation inducing tissue damage, persistent infection, and inhibition of epithelialization (Tomic‐Canic et al. 2020; Ramirez et al. 2018). DFUs can serve as entry points for microorganisms, including skin commensal bacteria that can colonize the ulcers and contribute to infection and delayed healing (Tomic‐Canic et al. 2020). Some potentially pathogenic bacteria form part of the microbiota that colonize the wound and are organized into biofilm consisting of a highly complex and organized polymicrobial community of bacteria and fungi surrounded by a self‐produced biopolymeric matrix. The biopolymeric matrix, made up of polysaccharides, proteins, lipids, and nucleic acids, protects the microorganisms from host responses and antibiotics (Tomic‐Canic et al. 2020; Yin et al. 2019).

To date, few therapeutic agents are available with US Food and Drug Administration (FDA) approval. These include recombinant PDGF‐BB and the skin substitutes Dermagraft™, Apligraf™, and Omnigraft™ (Bay et al. 2021; Sumpio et al. 2023). However, high failure rates are associated with these products. Furthermore, the use of topical antimicrobial agents as therapy is continuously debated (Lipsky et al. 2020).

Elucidating the cellular and molecular mechanisms involved in the development of DFUs and the role microorganisms play in DFU pathology is crucial for developing novel and effective treatment approaches. Traditionally, culture based‐methods have been used to determine the composition of bacteria present in skin microbiota and DFUs. Characterizing the full spectrum of polymicrobial community by these methods is challenging because of their laborious and time‐consuming nature making timely and accurate identification of microorganisms difficult. Also, they have low sensitivity and are unable to detect slow‐growing fastidious microorganims that may be prominent or clinically relevant (Sadeghpour Heravi et al. 2019; Travis et al. 2020). Recent advances in molecular microbiological techniques have addressed some of these limitations. Next‐generation sequencing does not require cultivation, exhibits greater sensitivity and allows for the identification and phylogenetic delineation of both the cultivable and uncultivable microbiome rapidly and comprehensively (Gardner et al. 2013; Zou et al. 2020).

In addition to culture‐based methods previous studies have used next‐generation DNA sequencing targeting the 16S ribosomal RNA (rRNA) gene and internal transcribed spacer (ITS) region of rRNA, to characterize the skin microbiome in patients with and without diabetes and the microbiome of DFUs (Travis et al. 2020; Gardner et al. 2013; Anafo et al. 2021; Han et al. 2020; Huang et al. 2022; Kalan et al. 2016; Makeri et al. 2024; Misic et al. 2014; Redel et al. 2013; Wolcott et al. 2016; Zhang et al. 2023; Ogai et al. 2022). Gardner et al. (2013) profiled the microbiome from neuropathic nonischemic DFU swabs utilizing next‐generation sequencing of the bacterial 16S rRNA gene and found an abundance of *Staphylococcus*, in particular *S. aureus* (Gardner et al. 2013). Another study profiled microorganisms from healthy skin, diabetic skin and DFU swabs using 16S rRNA gene sequencing and reported changes in the abundance of *Staphylococcus*, *Enhydrobacter, Corynebacterium, Escherichia coli*, and *Pseudomonas* in the skin bacterial colonies between these samples (Zhang et al. 2023). In a large‐scale retrospective study of the microbiota from chronic wound samples including DFUs, venous leg ulcers, decubitus ulcers and nonhealing surgical wounds the presence of *Staphylococcus* and *Pseudomonas* genera in 63% and 25% of wounds, respectively was reported. *S. aureus* and *S. epidermidis* were the predominant species, each comprising ca. 25% of the *Staphylococcus* strains identified in the wounds. *Pseudomonas* spp. were present in 25% of wound samples and exhibited the tendency to constitute a high proportion of the biofilm communities in which they were present (Wolcott et al. 2016). Commensal bacteria including *Corynebacterium* and *Propionibacterium* species and anaerobic bacteria were found to be prevalent in chronic wounds (Wolcott et al. 2016). Furthermore, a longitudinal study that explored the dynamic diversity of DFU mycobiome using high‐throughput sequencing of the rRNA ITS1 identified that the two most abundant species within the *Ascomycota* phylum were *Cladosporium herbarum* and *Candida albicans*, and the most abundant species within the *Basidiomycota* phylum were *Trichosporon* and *Rhodosporidium*, which are opportunistic yeast pathogens (Kalan et al. 2016). However, the majority of these next‐generation sequencing studies have not focused on Afro‐Caribbean populations, resulting in a scarcity of skin and DFU microbiome data for this group.

The economic, health‐related and quality of life impacts of DFUs necessitate comprehensive characterization of the microbiome inhabiting chronic DFUs. These efforts will be instrumental for discovering innovative treatment modalities which may alleviate these impacts. Hence, in the present pilot study we characterize the bacterial and fungal microbiome of tissue biopsies from chronic DFUs and matched healthy intact skin in Afro‐Caribbean patients with type 2 diabetes in Barbados using culture‐independent Illumina amplicon sequencing targeting the 16S rRNA gene and the internal transcribed spacer (ITS2) region of rRNA, respectively.

## 2 Materials and Methods

### 2.1 Participants Recruitment

**Figure 1:**
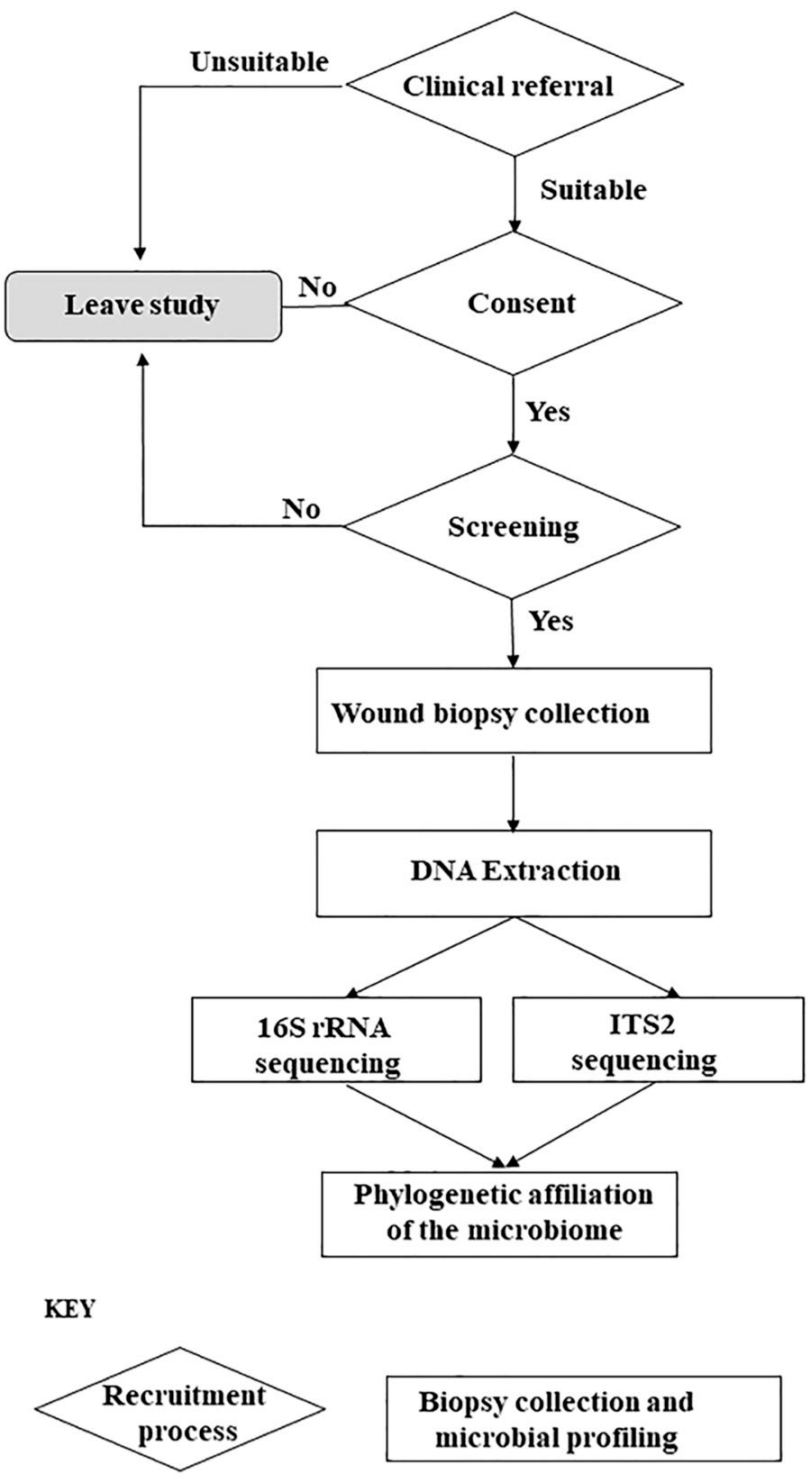
Study design: Participant flow from clinical referral through participant consent and screening to biopsy collection and microbiome profiling.

Patients with diabetes and foot ulcers were recruited from the surgical outpatient wound clinic of the Queen Elizabeth Hospital upon referral to the study by their consultant or wound clinic nurse. Once informed consent had been obtained (Figure 1), participants were screened for study inclusion and enrolled (Table 1). Patients who self‐identified as being of Afro‐Caribbean ethnicity, had a diagnosis of type‐2 diabetes, were 18 years of age or older, of either sex, and were capable of giving informed consent were eligible, provided that they had chronic, nonhealing diabetic foot ulcers (DFUs). Chronic, non‐healing DFUs were defined as those of more than 4 weeks' duration at the time of the study that had not responded to routine care, including offloading and revascularization (Table 1).

**Table 1:** Inclusion and exclusion criteria for study enrollment.

Inclusion criteria	Exclusion criteria
Age ≥ 18 years	Patients receiving systemic or topic antimicrobials or anti‐fungal treatment within two weeks of the study
Afro‐Caribbean origin	The clinician deems it inappropriate to take a tissue sample for any reason
Both sexes	DFU ≥ 1 week and ≤ 4 weeks
Able to give informed consent	Patient refusal
Current or previous diagnoses of type 2 diabetes	
Diabetic foot ulcer (DFU) clinically affected as per the University of Texas Wound classification System (Boulton and Whitehouse 2000) for diabetic wounds or	
Presence of chronic DFU (≥ 4‐weeks on enrollment) and not responding to routine care including offloading and revascularization	

### 2.2 Data Collection

On enrollment, demographic data, including date of birth, sex, age, and ethnicity, were collected. Health‐related information, such as pre‐existing conditions, diabetes medications, smoking status, and whether any systemic or topical antibiotics or antifungal treatments had been taken in the preceding 2 weeks, was also recorded. The DFU assessment was made by a physician, and information on its location, wound size, tissue type in the base of the ulcer, and severity of the wound using the Texas wound classification system (Boulton and Whitehouse 2000) was collected.

### 2.3 Tissue Biopsy Collection

Once eligibility was confirmed, DFUs were cleaned with sterile saline and gauze. For each patient, one 3 mm wide × 10 mm deep tissue punch biopsy from the center of the ulcer was taken and after aseptic removal were briefly washed with sterile saline (0.85% NaCl) solution to remove coagulated blood. A second 3 mm wide × 10 mm deep tissue punch biopsy of clinically normal, intact skin was taken at approximately 1‐cm from the wound edge of the same foot and washed. This position allowed for the control site to be close enough to represent the same local microenvironment so that anatomical location, laterality, vascular supply, neuropathic status and environmental exposure were comparable, while being sufficiently distant from the ulcer to minimize any direct microbial spillover to the normal skin. Thus, each participant served as his own matched control. The anatomical locations of the DFUs are listed in Table 2. All biopsy specimens were placed in separate sterile containers, stored at −20°C in the hospital, transported on ice, and stored at −20°C in the laboratory until DNA extraction.

**Table 2:** Patient demographics and wound characteristics.

Patient	Sex	Age	Ulcer duration	Wound size (Depth [mm] × Width [mm])	DFU location	DFU neuropathic?	The University of Texas wound classification system
1 (DCP‐05)	Male	55	3 months	5 mm × 30 mm	Poly digits	No	A1
2 (DCP‐07)	Male	73	6 weeks	5 mm × 45 mm	Medial forefoot	No	A1
3 (DCP‐08)	Male	60	8 months	2 mm × 5 mm	Medial forefoot	Yes	A1
4 (DCP‐11)	Male	48	3 years	5 mm × 80 mm	Dorsum midfoot	No	A1
5 (DCP‐14)	Male	54	3 months	30 mm × 70 mm	Poly digits	No	C3
6 (DCP‐15)	Male	53	4 months	5 mm × 40 mm	Plantar forefoot	No	A1
7 (DCP‐16)	Male	55	12 months	5 mm × 10 mm	Plantar forefoot	Yes	A3
8 (DCP‐18)	Male	49	12 months	20 mm × 35 mm	Plantar midfoot	Yes	A1

Abbreviations: DCP, patients with diabetes from outpatients' wound clinic; DFU, diabetic foot ulcer.

### 2.4 DNA Extraction and Illumina Amplicon Sequencing

Each biopsy was homogenized with sterile scalpel and the homogenates (ca. 0.2 g) were used for the extraction of genomic DNA using the SurePrep™ soil DNA isolation kit (Thermo Fisher Scientific, Fair Lawn, NJ) as per manufacturer's instructions. Extracted DNA was eluted in the elution buffer supplied in the kit and stored at −20°C. DNA concentration was quantified utilizing QuantiFluor™ ONE dsDNA System (Promega, Madison, WI) and Quantus™ Fluorometer (Promega, Madison, WI). The quality of the extracted DNA samples was assessed using the NanoDrop spectrophotometer (Thermo Fisher Scientific, Fair Lawn, NJ).

The extracted DNA from biopsies was sequenced using the Illumina MiSeq platform with a 250 bp paired‐end library preparation protocol (MiSeq Reagent Kit V2) (Illumina, San Diego, CA). Bacterial communities were profiled by targeting the V3‐V4 region of the 16S rRNA gene using primers Illumina‐F (5′‐TCGTCGGCAGCGTCAGATGTGTATAAGAGACAGCCTACGGGNGGCWGCAG‐3′) and Illumina‐R (5′‐ GTCTCGTGGGCTCGGAGATGTGTATAAGAGACAGGACTACHVGGGTATCTAATCC‐3′), comprising Illumina adapter overhangs appended to the gene‐specific sequences of Klindworth et al. (2012), as described in the Illumina 16S Metagenomic Sequencing Library Preparation protocol (Illumina 2013). Fungal communities were profiled by targeting the ITS2 region using primers ITS1F (5′‐CTTGGTCATTTAGAGGAAGTAA‐3′) and ITS4 (5′‐TCCTCCGCTTATTGATATGC‐3′) (Gardes and Bruns 1993; White et al. 1990).

### 2.5 Bioinformatic Processing and Taxonomic Assignment

The raw reads of 16S rRNA gene and ITS2 were processed via EzBioCloud Microbial Taxonomic Profiling (MTP) pipeline (Yoon et al. 2017) by merging the paired‐end reads and filtering the low‐quality reads. The quality‐controlled reads were further processed via MTP pipeline for denoising and extraction of non‐redundant reads, removing chimeric sequences, and picking Operational Taxonomic Unit (OTUs) at 97% sequence similarity. The EzBioCloud 16S (PKSSU 4.0) and the ITS2 (FGITS 1.0) databases were then used to taxonomically assign OTUs using standard similarity thresholds as implemented in the validated EzBioCloud MTP pipeline, as per published guidelines of ITS2‐based fungal profiling.

### 2.6 Diversity Analyses

The estimation of alpha diversity: Species richness: ACE (Chao and Lee 1992), Chao1 (Chao 1984), Jackknife (Burnham and Overton 1978, 1979), number of OTUs; Diversity index: Shannon (Shannon 1948), Simpson (Simpson 1949); Species evenness: Pielou's evenness (Pielou 1966); Coverage of data: (Good's coverage of library (%)) (Good 1953), and phylogenetic diversity (equivalent to Faith's Phylogenetic diversity [Faith 1992]) and beta diversity (Bray‐Curtis dissimilarity) (Bray and Curtis 1957) were computed using the EzBioCloud MTP pipeline (Yoon et al. 2017). Bray–Curtis dissimilarity matrices were ordinated using principal coordinates analysis (PCoA) and samples were hierarchically clustered using UPGMA (average‐linkage) clustering based on Bray–Curtis dissimilarities.

### 2.7 Statistical Analyses

Beta diversity was statistically evaluated via beta set‐significance analysis using permutational multivariate analysis of variance (PERMANOVA) (Anderson 2001). The Wilcoxon Signed‐Rank test (two‐sided), a non‐parametric test appropriate for small, paired samples with non‐normally distributed data, was used to statistically compare the alpha diversity indices between DFUs and matched control samples, reflecting the within‐patient matched design. The Wilcoxon Signed‐Rank test was also used to compare sequencing output/quality summary metrics between matched DFUs and controls in the sequencing information for bacterial and fungal communities. All tests were two‐tailed with no a priori directional hypothesis specified. A *p*‐value of < 0.05 was considered statistically significant. Statistical analyses were conducted using R (version 4.x; R Core Team) (R Core Team 2026).

## 3 Results

Eight patients participated in the study. Their demographics are summarized in Table 2. All participants were Afro‐Caribbean male patients, aged between 48 and 73 years old, with type 2 diabetes. They presented with ulcers at different foot locations, with ulcer duration ranging from 6 weeks to 3 years. Three out of the eight patients had neuropathic diabetic foot ulcers (DFUs).

### 3.1 Sequence Data

The sequence information including paired‐end raw sequences, low quality amplicons, nontarget amplicons, chimeric amplicons, total valid reads, unique sequences (OTUs) and average base pairs (bp) for prokaryotic and fungal communities found in DFUs and control samples are included in Tables 3 and 4, respectively. Controls and DFUs were profiled and a total of 1,259,000 and 200,111 paired‐end raw sequences were obtained through targeting of the V3‐V4 region of the 16S rRNA gene and the ITS2 region of the rRNA, respectively, from control samples (566,228 [16S rRNA] and 105,609 [ITS2] of the rRNA) and DFUs (692,772 [16S rRNA] and 94,502 [ITS2]). After quality filtering, including trimming, paired‐end reads merging, and removal of low quality, non‐target and chimeric amplicons, the total valid reads for 16S rRNA genes were 471,276 for control samples compared to 609,289 for DFUs with no significant difference observed (Wilcoxon signed‐rank test, W = 4.0, *p* = 0.055). The total reads for the ITS2 region of the rRNA operon were 48,179 for controls and 45,723 for DFUs with no significant difference observed (Wilcoxon signed‐rank test, W = 12.0, *p* = 0.461). An average size of 419 bp (controls: 418 bp; DFUs: 420 bp) and 331.5 bp (controls: 322 bp; DFUs: 341 bp) for V3‐V4 and ITS2 regions, respectively, were kept. These sequences of V3‐V4 and ITS2 regions were categorized into a combined 1296 (controls: 629, DFUs: 667) and 5079 (controls: 2674, DFUs: 2405) operational taxonomic units (OTUs), respectively with no significant differences observed between control samples and DFUs in the retained sequences of V3‐V4 (Wilcoxon signed‐rank test, W = 12.0, *p* = 0.813) or the ITS2 regions (Wilcoxon signed‐rank test, W = 6.0, *p* = 0.102) (Tables 3 and 4).

**Table 3:** Sequences information for bacterial communities in DFUs and controls.

Sample name	Paired‐end raw sequences	Low quality amplicons	Nontarget amplicons	Chimeric amplicons	Total valid reads	Unique sequences (OTUs)	Average bp
Control							
DCP‐053	64,276	1176	0.00	14,766	48,334	78	414.3
DCP‐073	83,896	6462	0.00	5587	71,847	81	419
DCP‐083	84,628	1507	0.00	18,700	64,421	113	406.4
DCP‐113	75,063	21,211	64.0	251	53,537	50	425.3
DCP‐143	87,411	2818	0.00	1306	83,287	34	408.1
DCP‐153	19,288	10,443	6.00	53	8786	101	422.9
DCP163	58,793	2866	5.00	653	55,269	95	423.9
DCP‐183	92,873	2806	8.00	4264	85,795	77	421.3
Total	**566,228**	**49,289**	**83.0**	**45,580**	**471,276**	**629**	**3341.2**
Mean	**70,779**	**6161**	**10.4**	**5698**	**58,910**	**78.6**	**418**
Wound							
DCP‐054	77,051	1343	0.00	10,203	65,505	57	414
DCP‐074	89,651	3600	0.00	11,101	74,950	96	422
DCP‐084	79,655	1444	0.00	12,466	65,745	88	418.1
DCP‐114	78,032	3215	0.00	8223	66,594	73	425.6
DCP‐144	75,703	1813	0.00	5760	68,130	34	419.2
DCP‐154	79,475	3901	1.00	3420	72,153	152	423.7
DCP‐164	79,247	1861	5.00	3222	74,159	104	415.3
DCP184	133,958	3788	6.00	8111	122,053	63	421.2
Total	**692,772**	**20,965**	**12.00**	**62,506**	**609,289**	**667**	**3359**
Mean	**86,597**	**2621**	**1.50**	**7813**	**76,161**	**83.4**	**420**
*p*‐value ≤ 0.05					**0.055**	**0.813**	

Abbreviations: DCP, patients with diabetes from outpatients' wound clinic; DFU, diabetic foot ulcer.

**Table 4:** Sequences Information for fungal communities in DFUs and controls.

Sample name	Paired‐end raw sequences	Low quality amplicons	Nontarget amplicons	Chimeric amplicons	Total valid reads	Unique sequences (OTUs)	Average bp
Controls							
DCP‐053	4714	1092	1647	0	1975	244	327.7
DCP‐073	4245	1295	1176	0	1774	138	332.1
DCP‐083	5564	1073	1208	3	3280	347	271
DCP‐113	3104	623	1128	8	1345	172	350.3
DCP‐143	1473	379	553	0	541	109	337.8
DCP‐153	31,876	8237	10,158	13	13,468	536	336.3
DCP‐163	11,133	2059	3341	0	5733	359	300.9
DCP‐183	43,500	8102	15,335	0	20,063	769	320.2
Total	**105,609**	**22,860**	**34,546**	**24**	**48,179**	**2674**	**2576.3**
Average	**13,201**	**2858**	**4318**	**3**	**6022**	**334**	**322**
Wound							
DCP‐054	3347	831	1030	7	1479	191	317.4
DCP‐074	6769	1750	1497	0	3522	151	368.6
DCP‐084	5673	899	748	25	4001	278	356.6
DCP‐114	1740	372	681	0	687	109	351.1
DCP‐144	680	205	211	0	264	56	325.6
DCP‐154	28,342	5843	9896	0	12,603	593	356.1
DCP‐164	7414	1541	2312	0	3561	283	311.1
DCP‐184	40,537	5038	15,893	0	19,606	744	341.2
Total	**94,502**	**16,479**	**32,268**	**32**	**45,723**	**2405**	**2727.7**
Average	**11,813**	**2060**	**4034**	**4**	**5715**	**301**	**341**
*p*‐value ≤ 0.05					**0.461**	**0.102**	

Abbreviations: DCP, patients with diabetes from outpatients' wound clinic; DFU, diabetic foot ulcer.

### 3.2 Bacterial and Fungal Diversity in Controls and DFUs

#### 3.2.1 Alpha Diversity

The alpha‐diversity indices for bacteria are included in Supporting Information: Table S1 and for fungi, are included in Supporting Information: Table S2. For bacteria, the comparative mean ± SD values for species richness (ACE, Chao1, Jackknife, the number of OTUs) recorded for controls were 97.4 ± 41.8, 93.5 ± 38.2, 96.5 ± 38.6, and 78.6 ± 26.1, respectively, and for DFUs 94.8 ± 48.8, 91.9 ± 25.4, 97.1 ± 48.1, and 83.4 ± 35.8, respectively. The Shannon and Simpson diversity indices (mean ± SD) values were estimated for controls as 1.64 ± 0.57 and 0.34 ± 0.22, respectively, and for DFUs 1.84 ± 0.36 and 0.24 ± 0.07, respectively. Phylogenetic diversity was 160.5 ± 73.3 for controls and 156.6 ± 87.0 for DFUs. The Pielou's index of species evenness (mean ± SD values) was recorded as 0.38 ± 0.13 and 0.42 ± 0.05 for controls and DFUs, respectively. The Good's coverage of library values was estimated as 100% both for the controls and DFUs. The Wilcoxon Signed‐Rank tests (paired, *n* = 8) showed no statistically significant differences in any bacterial alpha diversity metric between controls and DFUs, including species richness (OTUs; W = 12.0, *p* = 0.813), Shannon diversity (W = 9.0, *p* = 0.250), Simpson index (W = 10.0, *p* = 0.313), Pielou's evenness (W = 9.0, *p* = 0.250), and phylogenetic diversity (W = 15.0, *p* = 0.719) (Supporting Information: Tables S1 and S3). Phylogenetic diversity showed inter‐patient variability highlighting the heterogeneous nature of the DFU microbiomes. Taken collectively, the data suggests a comparable bacterial phylogenetic diversity between the ulcer samples and matched control samples.

For fungi, the comparative mean ± SD values for species richness (ACE, Chao1, Jackknife, the number of OTUs) recorded for controls were 337 ± 225, 334 ± 225, 338 ± 226, and 334 ± 225, respectively, and for DFUs 302 ± 243, 301 ± 243, 304 ± 244, and 301 ± 243, respectively. The Shannon and Simpson diversity indices (mean ± SD) values were estimated for controls as 4.29 ± 0.29 and 0.04 ± 0.01, respectively, and for DFUs 3.91 ± 0.54 and 0.07 ± 0.05, respectively. Phylogenetic diversity was 173.1 ± 42.7 for controls and 156.9 ± 58.6 for DFUs. The Pielou's index of species evenness (mean ± SD values) was recorded as 0.77 ± 0.06 and 0.73 ± 0.11 for controls and DFUs, respectively. The Good's coverage of library values was estimated as 99.8% both for the controls and DFUs. The Wilcoxon Signed‐Rank tests (paired, *n* = 8) revealed that fungal Shannon diversity was significantly lower in DFUs compared to controls (W = 3.0, *p* = 0.039). Phylogenetic diversity displayed a trend towards reduced values in DFUs (W = 5.0, *p* = 0.078), consistent with the observed reduction in Shannon diversity. No significant differences were observed for other fungal alpha diversity metrics, including OTU richness (W = 6.0, *p* = 0.102), Simpson index (W = 3.0, *p* = 0.156), and Pielou's evenness (W = 14.0, *p* = 0.641) (Supporting Information: Tables S2 and S4). Collectively, the data suggests that in DFU wounds, fungal diversity is reduced.

### 3.3 Beta Diversity

**Figure 2:**
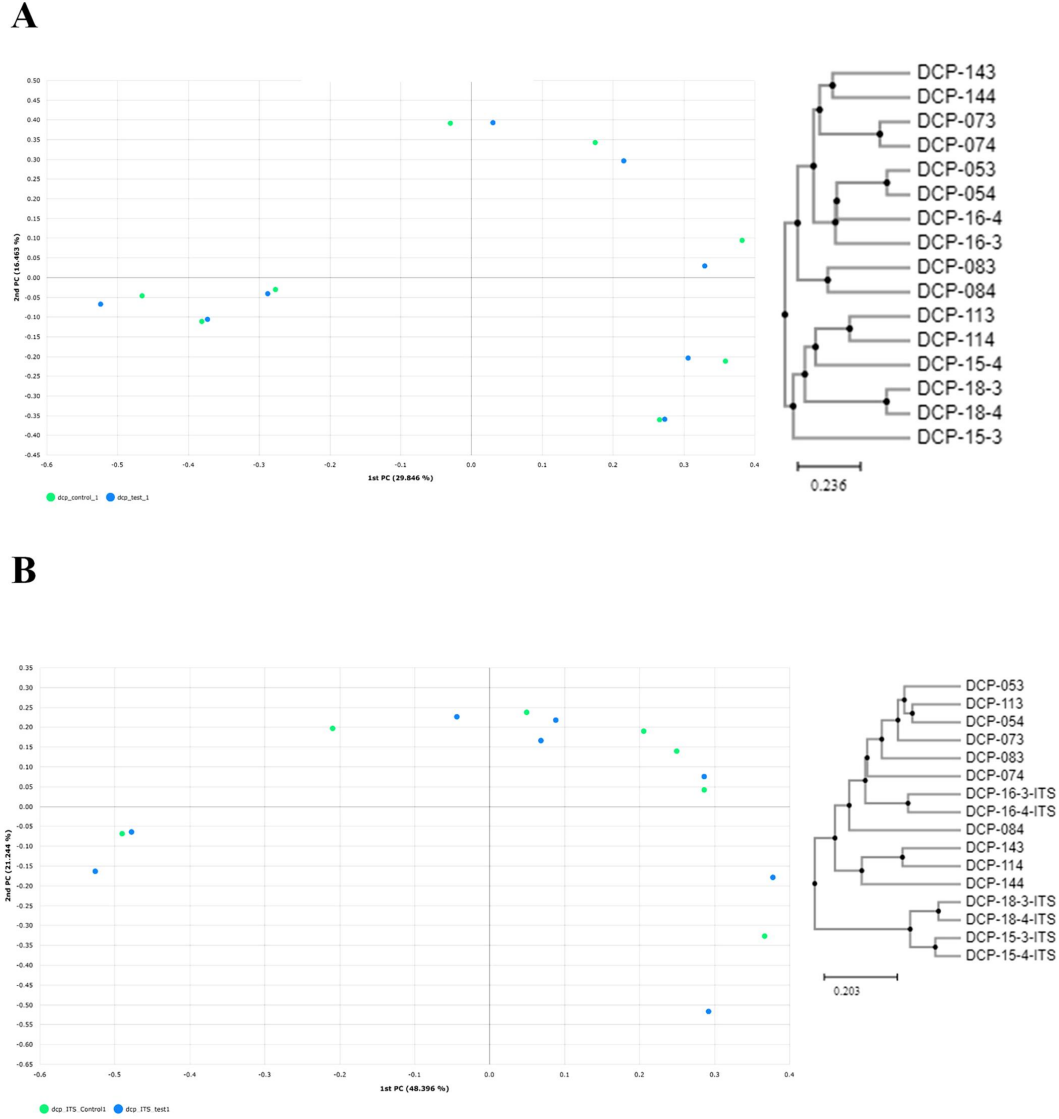
Bray–Curtis PCoA of bacterial and fungal communities in DFUs and controls. Beta diversity of (A) bacterial (16S rRNA) and (B) fungal (ITS2) communities in DFUs (DCP‐054, 074, 084, 114, 144, 15‐4, 16‐4, 18‐4) (*n* = 8; blue) and controls (DCP‐053, 073, 083, 113, 143, 15‐3, 16‐3, 18‐3) (*n* = 8; green). PCoA plots based on Bray–Curtis dissimilarities are shown on the left, with the percentage of variation explained by each axis indicated (bacteria: PC1 = 29.846%, PC2 = 16.463%; fungi: PC1 = 48.396%, PC2 = 21.244%). UPGMA clustering dendrograms based on Bray–Curtis dissimilarities are shown on the right. Bray–Curtis dissimilarities were computed in EzBioCloud using the “Species” profile with unclassified OTU reads included. Group differences were assessed using PERMANOVA on Bray–Curtis dissimilarities (bacteria: *p* = 0.982; Supporting Information: Table S5; fungi: *p* = 0.975; Supporting Information: Table S6). DFUs, diabetic foot ulcers; ITS2, internal transcribed spacer 2; OTU, operational taxonomic unit; PCoA, principal coordinates analysis; PERMANOVA, permutational multivariate analysis of variance; UPGMA, unweighted pair group method with arithmetic mean.

Beta diversity was assessed using Bray–Curtis dissimilarity. For bacterial community, mean Bray–Curtis distances for controls and DFUs were recorded as 0.997 and 0.998, respectively (Supporting Information: Table S5), and for fungal community, mean Bray–Curtis distances for controls and DFUs were recorded as 0.949 and 0.976, respectively (Supporting Information: Table S6). Beta diversity based on Bray–Curtis dissimilarities showed no evidence of overall community composition differences between DFUs and controls for bacteria (PERMANOVA *p* = 0.982; Supporting Information: Table S5) or fungi (PERMANOVA *p* = 0.975; Supporting Information: Table S6). PCoA plots based on Bray–Curtis dissimilarity (Figure 2) showed intermixing and overlap between DFU and control samples for both prokaryotic (Figure 2A) and fungal (Figure 2B) communities and revealed no clear separation in overall community composition between the two groups.

### 3.4 Bacterial and Fungal Taxonomy in Controls and DFUs

#### 3.4.1 Bacteria

**Figure 3:**
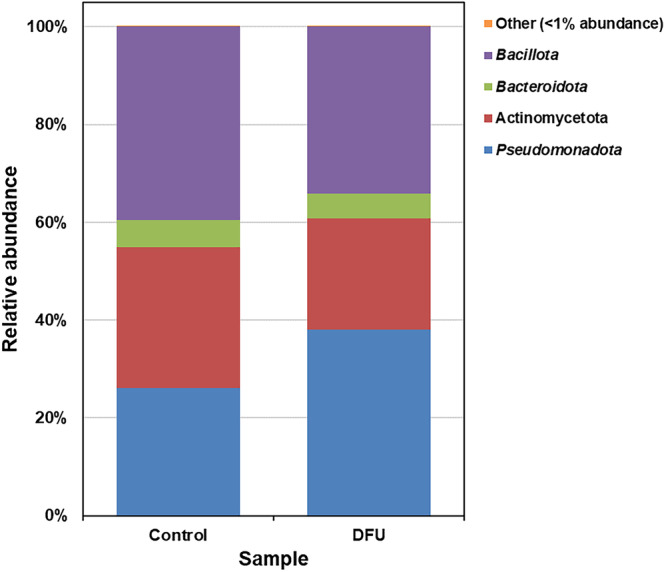
Taxonomic composition of bacterial community in DFUs and controls at phylum level. DFUs, diabetic foot ulcers.

**Figure 4:**
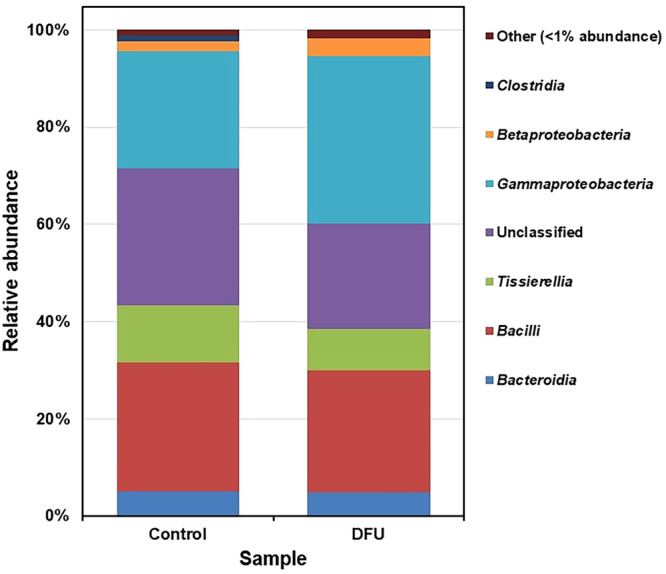
Taxonomic composition of bacterial community in DFUs and controls at class level. DFUs, diabetic foot ulcers.

**Figure 5:**
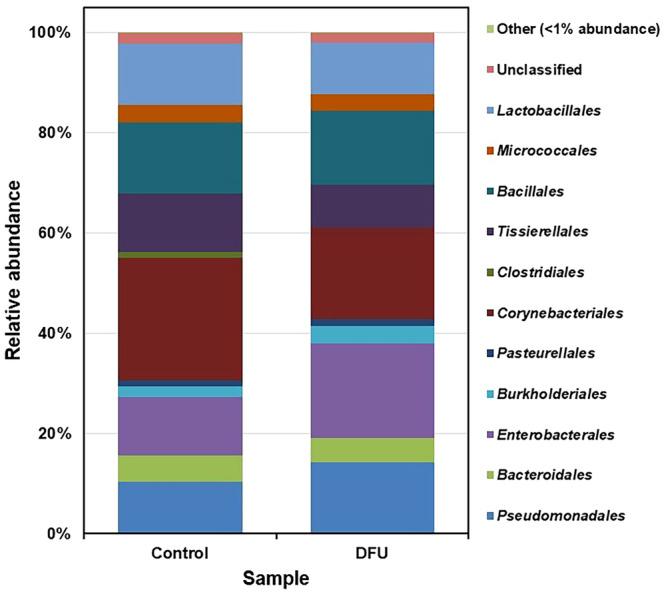
Taxonomic composition of bacterial community in DFUs and controls at order level. DFUs, diabetic foot ulcers.

**Figure 6:**
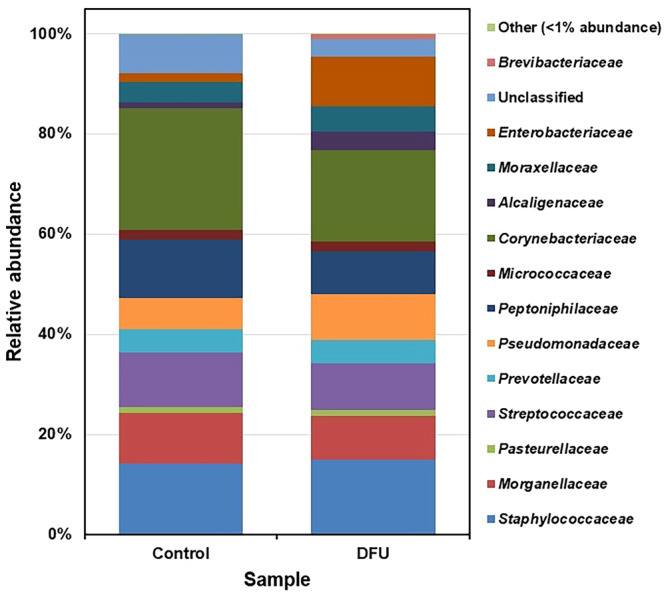
Taxonomic composition of bacterial community in DFUs and controls at family level. DFUs, diabetic foot ulcers.

**Figure 7:**
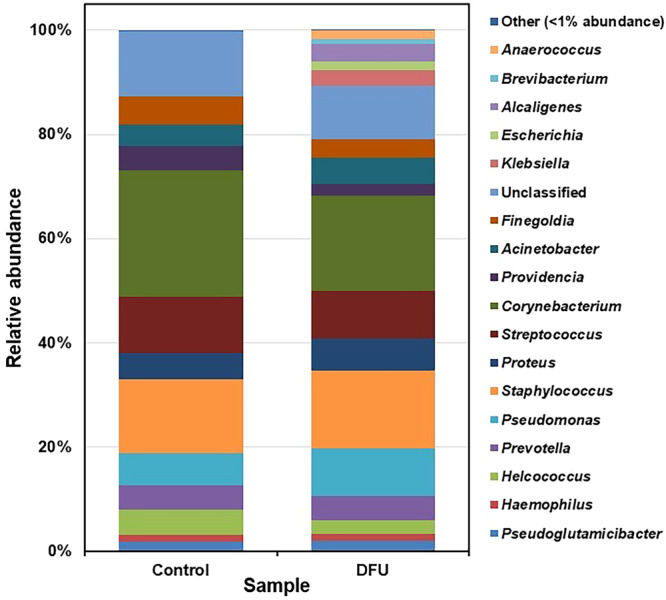
Taxonomic composition of bacterial community in DFUs and controls at genus level. DFUs, diabetic foot ulcers.

**Figure 8:**
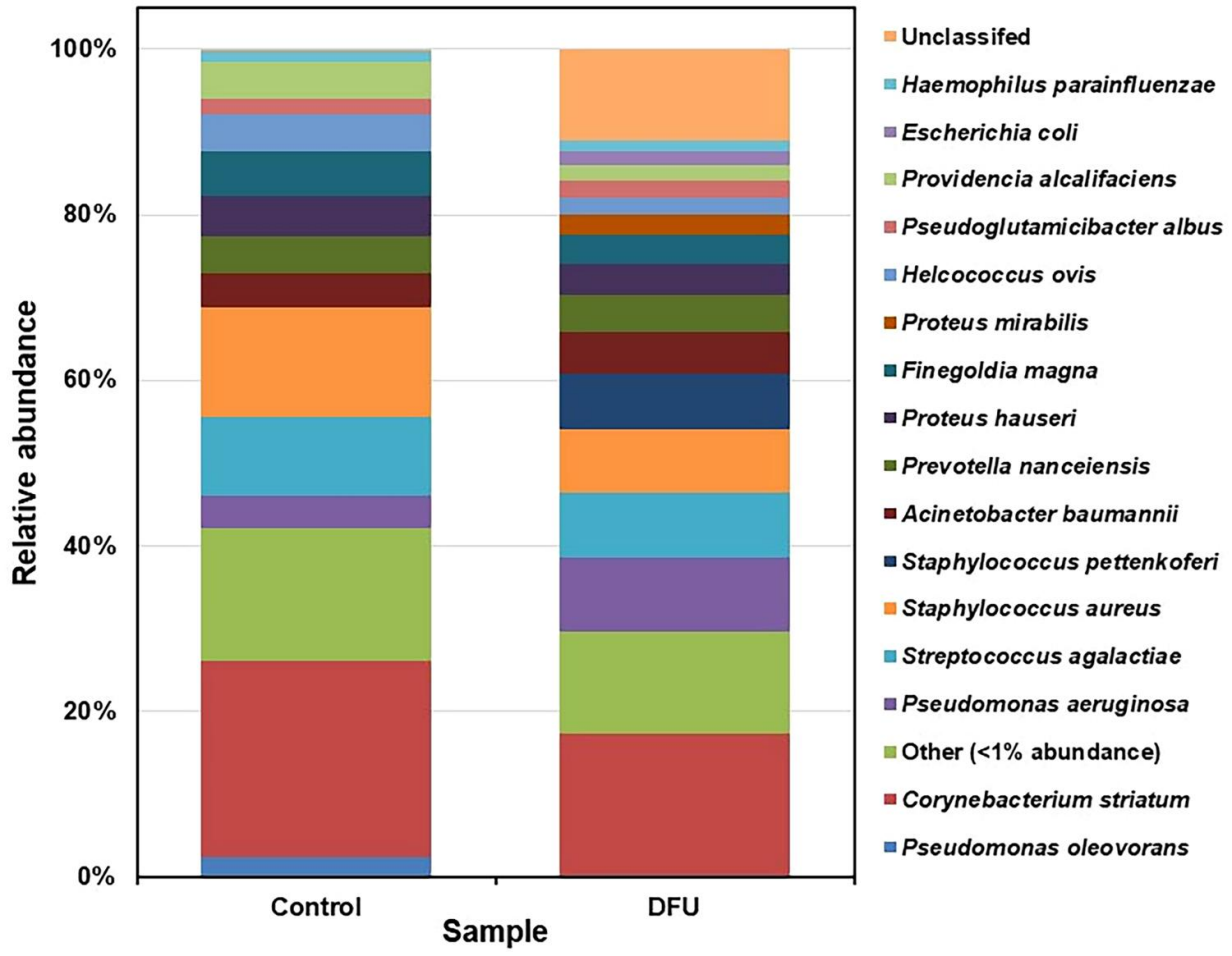
Taxonomic composition of bacterial community in DFUs and controls at species level. DFUs, diabetic foot ulcers.

The taxonomic compositions of bacterial communities, such as phylum, class, order, family, genus and species, in DFUs and controls are shown in Figures 3, 4, 5, 6, 7, 8. All OTUs were classified and categorized from DFUs and controls. At the phylum level, bacteria present in DFUs with a relative abundance > 1% were *Pseudomonadota* (formerly *Proteobacteria*) (38.1%), *Bacillota* (formerly *Firmicutes*) (34.2%), *Actinomycetota* (formerly *Actinobacteria*) (22.7%), and *Bacteroidota* (formerly *Bacteroidetes*) (5%). In comparison to the controls, the order of predominance were *Bacillota* (39.5%), *Actinomycetota* (28.8%), *Pseudomonadota* (26.2%) and of low relative abundance, *Bacteroidota* (5.5%) (Figure 3). At a relative abundance < 1%, six and eight phyla were identified in DFUs (total abundance 0.008%) and controls (total abundance 0.01%), respectively.

Five and six bacterial classes were detected in DFUs and controls, respectively, at > 1% relative abundance, with slightly different patterns observed, along with 21.8% and 28.2% of the unclassified classes, respectively. The top five classes present in DFUs were *Gammaproteobacteria* (34.4%), *Bacilli* (25.1%), *Tissierellia* (8.51%), followed by *Bacteroidia* (4.9%) and *Betaproteobacteria* (3.64%). In contrast, the six predominant classes identified in controls were *Bacilli* (26.5%), *Gammaproteobacteria* (24.3%), *Tissierellia* (11.8%), followed by *Bacteroidia* (5.18%), *Betaproteobacteria* (2.09%), and *Clostridia* (1.15%) (Figure 4). At a relative abundance < 1%, 15 classes each were identified in both DFUs (total abundance 1.71%) and controls (total abundance 1.11%).

Ten and 11 bacterial orders were detected in DFUs and controls, respectively, at > 1% relative abundance along with 2% and 2.1% of the unclassified orders, respectively. The most dominant bacterial orders recorded in DFUs were *Enterobacterales* (18.7%), *Corynebacteriales* (18.3%), followed by *Bacillales* (14.9%), *Pseudomonadales* (14.3%), *Lactobacillales* (10.2%), *Tissierellales* (8.5%), *Bacteroidales* (4.9%), *Burkholderiales* (3.6%), *Micrococcales* (3.4%), and *Pasteurellales* (1.3%). In controls, *Corynebacteriales* (24.3%) was also the most dominant, followed by *Bacillales* (14.2%), *Lactobacillales* (12.3%), *Tissierellales* (11.8%), *Enterobacterales* (11.7%), *Pseudomonadales* (10.4%), *Bacteroidales* (5.2%), *Micrococcales* (3.4%), *Burkholderiales* (2.1%), *Pasteurellales* (1.4%), and *Clostridiales* (1.2%) (Figure 5). At a relative abundance < 1%, 34 and 40 orders were identified in DFUs (total abundance 0.021%) and controls (total abundance 0.020%), respectively.

Thirteen and 12 bacterial families were detected in DFUs and controls, respectively, at > 1% relative abundance along with 3.5% and 7.8% of the unclassified families, respectively. At > 5% relative abundance, the predominant bacterial families present in DFUs were *Corynebacteriaceae* (18.3%), *Staphylococcaceae* (14.9%), *Enterobacteriaceae* (9.9%), *Streptococcaceae* (9.2%), *Pseudomonadaceae* (9.2%), *Morganellaceae* (8.8%), *Peptoniphilaceae* (8.5%), *Moraxellaceae* (5.1%), and in controls were, *Corynebacteriaceae* (24.3%), *Staphylococcaceae* (14.2%), *Peptoniphilaceae* (11.8%), *Streptococcaceae* (10.9%), *Morganellaceae* (10.1%), and *Pseudomonadaceae* (6.3%). At > 1% relative abundance, *Brevibacteriaceae* (1.1%) was present in DFUs but absent in controls (Figure 6). At a relative abundance < 1%, 71 and 77 families were identified in DFUs (total abundance 0.04%) and controls (total abundance 0.08%), respectively.

Seventeen and 12 bacterial genera were detected in DFUs and controls, respectively, at > 1% relative abundance along with 10.3% and 12.6% of the unclassified genera, respectively. At > 5% relative abundance, the predominant bacteria at the genus level identified in DFUs were *Corynebacterium* (18.3%), *Staphylococcus* (14.9%), *Streptococcus* (9.2%), *Pseudomonas* (9.2%), *Proteus* (6.2%), and *Acinetobacter* (5.1%) and in controls were, *Corynebacterium* (24.3%), *Staphylococcus* (14.1%), *Streptococcus* (10.9%), *Pseudomonas* (6.3%), *Finegoldia* (5.5%), and *Proteus* (5.1%). At > 1% relative abundance, the bacterial genera *Alcaligenes* (3.3%), *Klebsiella* (2.9%), *Escherichia* (1.7%), *Anaerococcus* (1.5%), and *Brevibacterium* (1.1%) were present in DFUs but absent in control samples (Figure 7). At a relative abundance < 1%, 130 and 150 genera were identified in DFUs (total abundance 0.09%) and controls (total abundance 0.14%), respectively.

Sixteen and 14 bacterial species were detected in DFUs and controls, respectively, at > 1% relative abundance along with 11.1% and 0.15% of the unclassified species, respectively. At > 5% relative abundance, the predominant bacterial species present in DFUs were *Corynebacterium striatum* (17.3%), *Pseudomonas aeruginosa* (8.9%), *Streptococcus agalactiae* (8%), *Staphylococcus aureus* (7.6%), *Staphylococcus pettenkoferi* (6.7%), and *Acinetobacter baumannii* (5.1%) and in controls were, *C. striatum* (23.8%), *S. aureus* (13.3%), *Streptococcus agalactiae* (9.5%) and *Finegoldia magna* (5.5%). At > 1% relative abundance, the species of *Staphylococcus pettenkoferi* (6.7%), *Proteus mirabilis* (2.5%), and *Escherichia coli* (1.73%) were all present in DFUs but absent in controls. However, the species of *P. oleovorans* (2.33%) was present in controls but absent in DFUs (Figure 8). At a relative abundance < 1%, 274 and 270 species were identified in DFUs (total abundance 12.3%) and controls (total abundance 15.9%), respectively.

#### 3.4.2 Fungi

**Figure 9:**
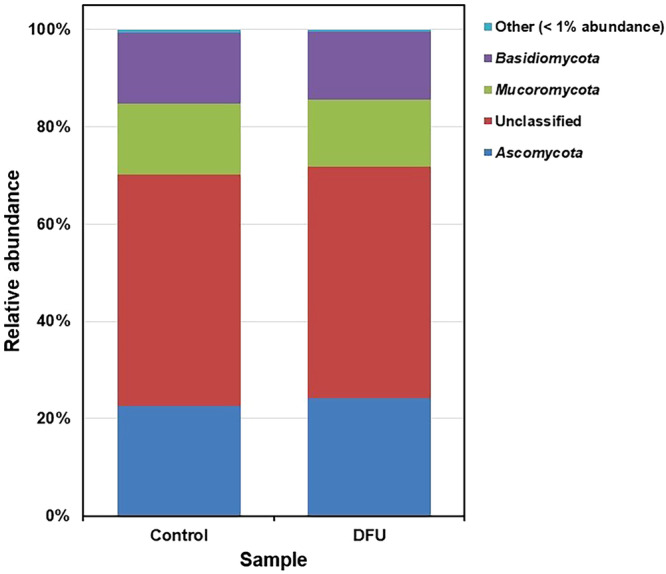
Taxonomic composition of fungal community in DFUs and controls at phylum level. DFUs, diabetic foot ulcers.

The 2405 and 2674 OTUs acquired via sequencing the ITS2 biomarker of DFUs and control samples, respectively, were assigned to 12 fungal phyla. The unclassified fungal populations at the phylum level at above 1% relative abundance were 47.6% for both DFUs and controls. The most dominant fungal phyla (> 1% relative abundance) that were detected in both DFUs and controls were *Ascomycota* (DFUs: 24.3%; controls: 22.6%), *Mucoromycota* (DFUs: 13.8%; controls: 14.7%) and *Basidiomycota* (DFUs: 13.8%; controls: 14.5%) (Figure 9). The other identified fungal phyla of low relative abundance (< 1%), *Rozellomycota, Kickxellomycota, Entomophthoromycota, Chytridiomycota, Mortierellomycota, Glomeromycota*, and *Neocallimastigomycota* were present in both DFUs and controls whereas *Entorrhizomycota* and *Calcarisporiellomycota* were absent in controls.

**Figure 10:**
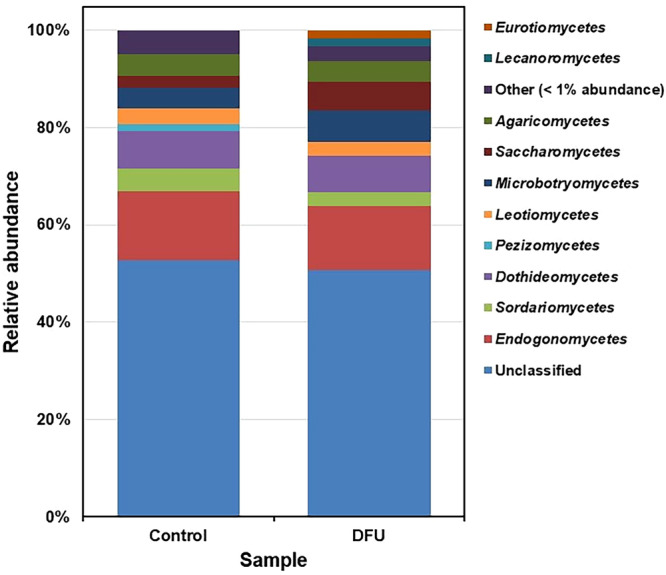
Taxonomic composition of fungal community in DFUs and controls at class level. DFUs, diabetic foot ulcers.

At the class level, the relative abundance above 1% of the unclassified fungal populations was recorded as 50.8% for DFUs and 52.7% for controls, indicating the presence of novel lineages. At > 5% relative abundance, the predominant fungal class identified in DFUs were *Endogonomycetes* (13.1%), *Dothideomycetes* (7.35%), *Microbotryomycetes* (6.44%), and *Saccharomycetes* (5.9%) and in controls were *Endogonomycetes* (14.1%) and *Dothideomycetes* (7.62%). At > 1% relative abundance, out of the 10 fungal class identified, two (*Lecanoromycetes* [1.65%] and *Eurotiomycetes* [1.62%]) were present in DFUs but absent in controls and one was present (*Pezizomycetes* [1.53%]) in controls but absent in DFUs (Figure 10). At a relative abundance < 1%, 12 and 16 classes were identified in DFUs (total abundance 3.14%) and controls (total abundance 4.98%), respectively.

**Figure 11:**
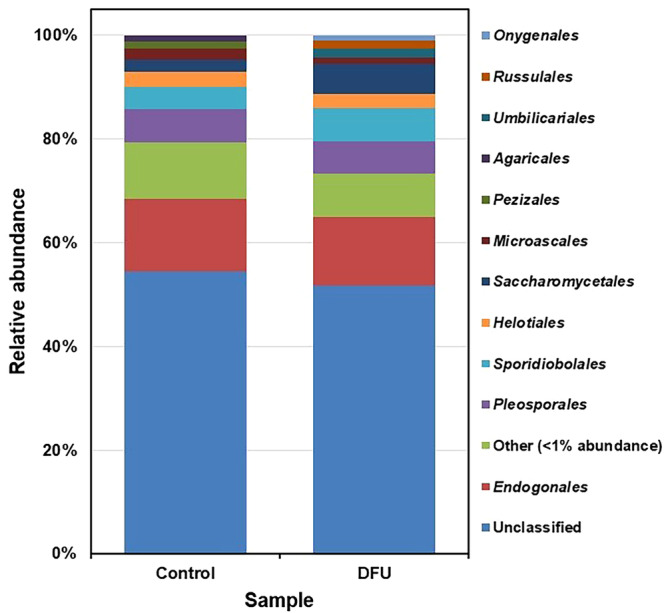
Taxonomic composition of fungal community in DFUs and controls at order level. DFUs, diabetic foot ulcers.

At the order level, the relative abundance above 1% of the unclassified fungal populations were recorded as 51.8% for DFUs and 53.8% for controls. At > 5% relative abundance, the predominant fungal orders identified in DFUs were *Endogonales* (13.1%), *Sporidiobolales* (6.41%), *Pleosporales* (6.25%), and *Saccharomycetales* (5.89%) and in controls were, *Endogonales* (13.9%) and *Pleosporales* (6.35%) in controls. At > 1% relative abundance, out of the 12 fungal orders detected, three were present in DFUs (*Russulales* [1.66%], *Umbilicariales* [1.65%], and *Onygenales* [1.01%]) but absent in controls. On the other hand, three were present in controls (*Agaricales* [1.29%], *Pezizales* [1.38%], and *Diaporthales* [1.06%]) but absent in DFUs (Figure 11). At a relative abundance < 1%, 29 and 35 orders were identified in DFUs (total abundance 8.36%) and controls (total abundance 10.7%), respectively.

**Figure 12:**
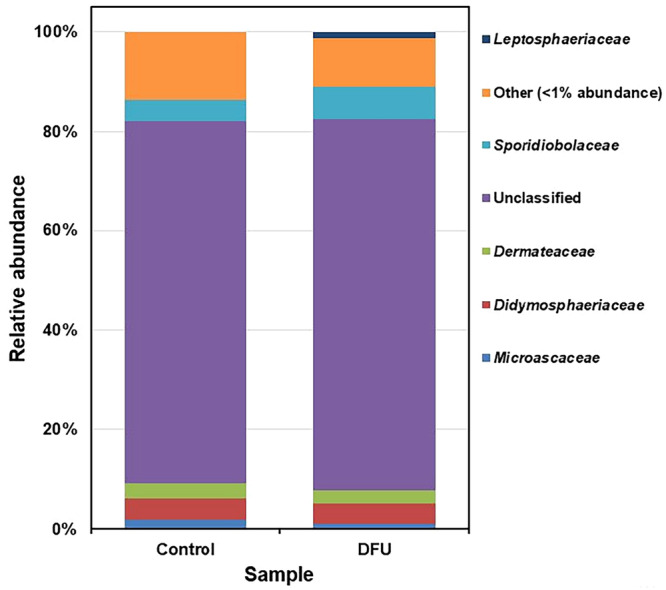
Taxonomic composition of fungal community in DFUs and controls at family level. DFUs, diabetic foot ulcers.

At the family level, the relative abundance above 1% of the unclassified fungal populations was recorded as 74.5% for DFUs and 73% for controls. At a relative abundance > 1%, five families were identified. The dominant fungal families present in DFUs were *Sporidiobolaceae* (6.38%), *Didymosphaeriaceae* (3.94%), *Dermateaceae* (2.66%), *Leptosphaeriaceae* (1.29%), and *Microascaceae* (1.18%). In controls, *Didymosphaeriaceae* (4.24%), *Sporidiobolaceae* (4.17%), *Dermateaceae* (2.90%), and *Microascaceae* (1.96%) were the most predominant (Figure 12). At a relative abundance < 1%, 45 and 49 families were identified in DFUs (total abundance 9.66%) and controls (total abundance 13.7%), respectively.

**Figure 13:**
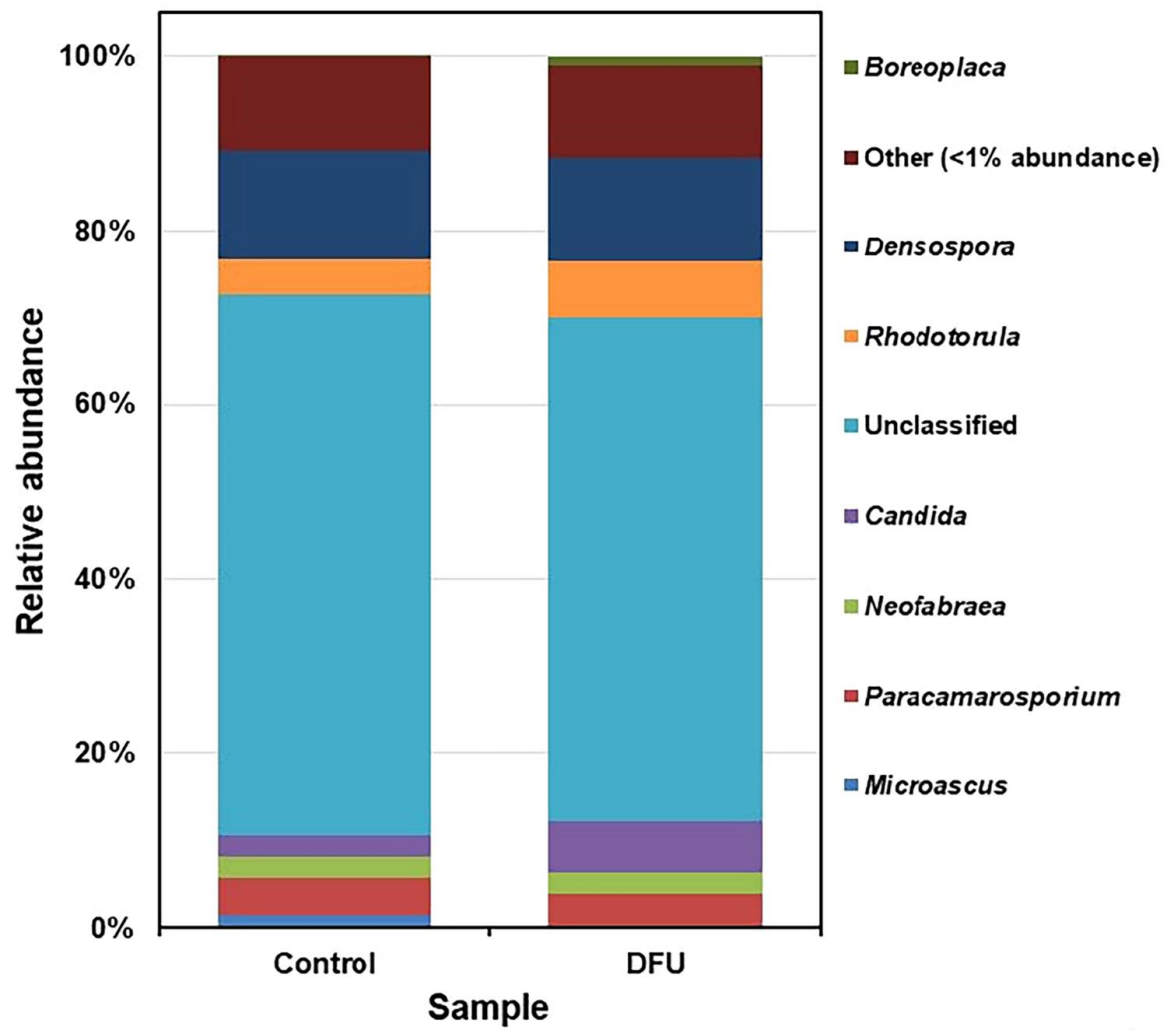
Taxonomic composition of fungal community in DFUs and controls at genus level. DFUs, diabetic foot ulcers.

At the genus level, the relative abundance above 1% of the unclassified fungal populations was recorded as 57.8% for DFUs and 61.9% for controls. At a relative abundance > 1%, the predominant fungal genera identified in DFUs were *Densospora* (12%), *Rhodotorula* (6.33%), *Candida* (5.8%), *Paracamarosporium* (3.94%), *Neofabraea* (2.42%), and *Boreoplaca* (1.05%). In controls, the predominant genera were *Densospora* (12.6%), *Paracamarosporium* (4.24%), *Rhodotorula* (4.06%), *Neofabraea* (2.45%), *Candida* (2.36%), and *Microascus* (1.58%) (Figure 13). At a relative abundance < 1%, 47 and 31 genera were identified in DFUs (total abundance 10.6%) and controls (total abundance 10.8%), respectively.

**Figure 14:**
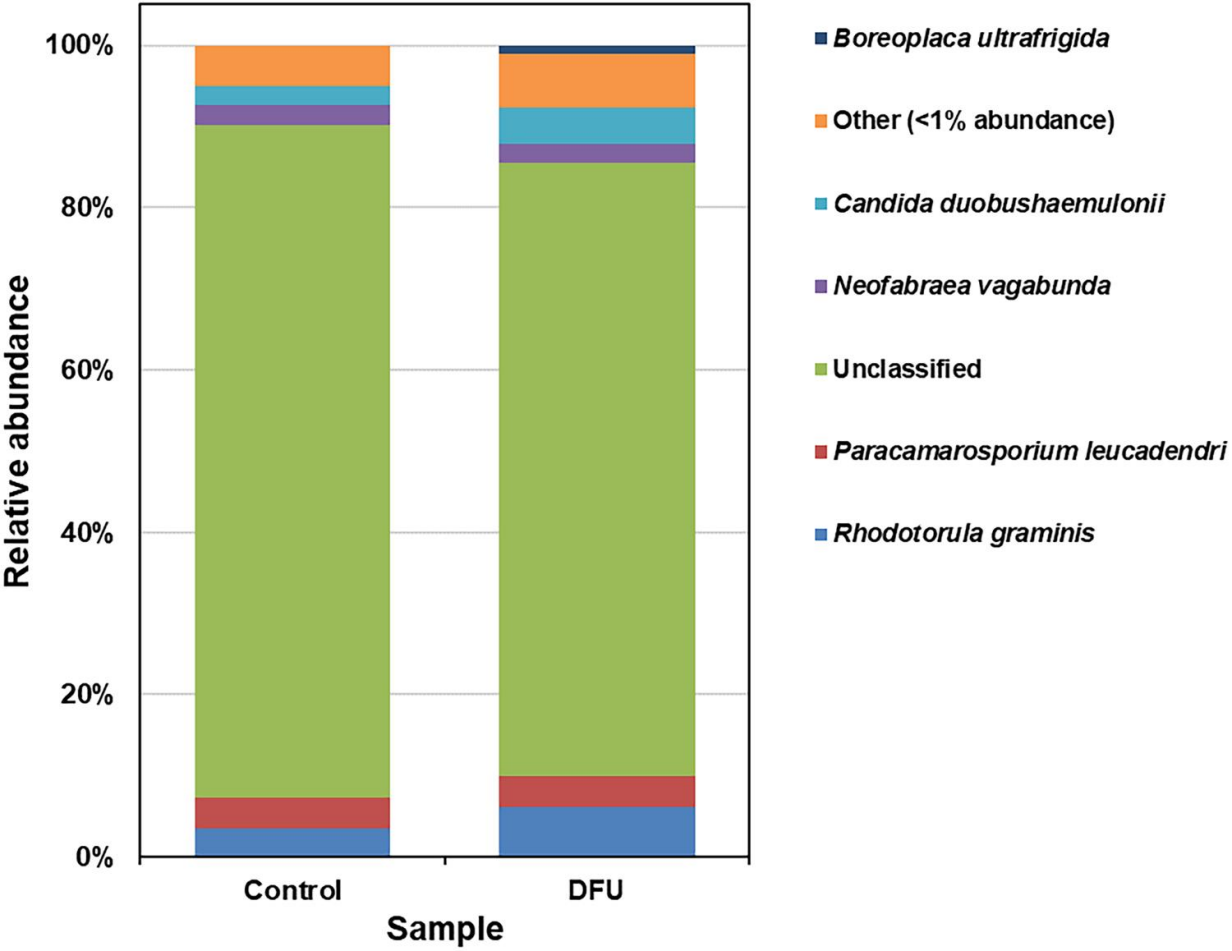
Taxonomic composition of fungal community in DFUs and controls at species level. DFUs, diabetic foot ulcers.

At the species level, the relative abundance above 1% of the unclassified fungal populations was recorded as 75.6% for DFUs and 82.9% for controls. At a relative abundance > 1%, five species were identified. The dominant fungal species present in DFUs were *Rhodotorula graminis* (6.18%), *Candida duobushaemulonii* (4.44%), *Paracamarosporium leucadendri* (3.77%), *Neofabraea vagabunda* (2.37%), and *Boreoplaca ultrafrigida* (1.03%). In controls, the order of predominance of species were *P. leucadendri* (3.85%), *R. graminis* (3.47%), *N. vagabunda* (2.38%), and *C. duobushaemulonii* (2.36%). At > 1% relative abundance, out of the five species detected, one was present in DFUs (*Boreoplaca ultrafrigida* [1.03%]) but absent in controls (Figure 14). At a relative abundance < 1%, 55 and 13 species were identified in DFUs (6.62%) and controls (5.03%), respectively.

## 4 Discussion

The skin microbiome comprises diverse communities including commensal fungi and bacteria that contribute to health, and during normal wound healing, are believed to play a key role in modulating the innate immune response (Tomic‐Canic et al. 2020). The colonization and transition of some of these opportunistic skin commensal microbes into pathogenic forms in DFUs and the formation of biofilms is thought to contribute to infection and play a role in the delay of wound healing (Tomic‐Canic et al. 2020; Kalan et al. 2019). To the best of the authors' knowledge, this pilot study provides the first characterization in Barbados of the bacterial and fungal taxonomic composition of chronic DFUs and matched intact skin in Afro‐Caribbean patients with type 2 diabetes. In this study, the alpha diversity indices suggested a diverse community of bacteria and fungi inhabiting Afro‐Caribbean patients with diabetes in both chronic ulcers (DFUs) and healthy intact skin (controls). The microbiome in DFUs and controls appeared to be similar, as demonstrated by the shared OTUs and similar predominance of the taxa of bacteria and fungi.

The alpha diversity analysis showed no statistically significant differences in bacterial community diversity between DFUs and controls. For fungal communities, Shannon diversity was lower in DFUs than in controls and this was statistically different (*W* = 3.0, *p* = 0.039), while the other fungal alpha diversity metrics showed no significant differences. Beta diversity analysis showed no major differences in overall community composition between DFUs and controls for either bacterial (*p* = 0.982) or fungal (*p* = 0.975) communities. PCoA plots based on Bray‐Curtis dissimilarity also displayed intermixing and overlap between DFU wounds and controls for both bacterial and fungal communities. The lack of significant differences in most diversity metrics is likely attributable to the paired within‐patient design of this study, in which DFU and matching control samples were taken from the same patient and local environment, as well as high degree of inter‐individual variability characteristic of small pilot studies of this kind. Though not statistically different, fungal phylogenetic diversity showed a directional trend towards lower values in DFUs compared to controls (*W* = 5.0, *p* = 0.078) similar to fungal Shannon diversity observed (*p* = 0.039). Taken together, this suggests a pattern of reduced fungal diversity in DFU wounds. This pattern is consistent with the rapid and dynamic changes reported in DFU mycobiome which correlates with delayed healing and poor outcome (Kalan et al. 2016); however, future work using larger longitudinal studies is warranted.

### 4.1 Bacteria

Gardner et al. (2013) profiled the microbiomes of swabs taken from neuropathic nonischemic DFUs without clinical evidence of infection using next‐generation sequencing (Gardner et al. 2013). Out of 13 phyla identified, the predominant sequences were classified to *Bacillota* (67%), *Actinomycetota* (14%), *Pseudomonadota* (9.8%), *Bacteroidota* (7.3%), and *Fusobacteriota* (formerly *Fusobacteria*) (1.4%) (Gardner et al. 2013). Our results are in agreement showing that the phyla of bacteria *Pseudomonadota*, *Bacillota*, *Actinomycetota*, and *Bacteroidota* were predominant in diabetic patients. However, in our study, *Pseudomonadota* (38.1%) was the predominant prokaryotic phylum present in DFUs whereas *Bacillota* (39.5%) was the predominant phylum found in controls. The difference in findings may be due to ethnicity, type of diabetes, DFU location, duration and grade, and biological sample used. In our study, tissue biopsies from both DFUs and adjacent healthy, undamaged skin were analyzed from eight Afro‐Caribbean male patients with type 2 diabetes at different foot locations, with ulcer duration ranging from 6 weeks to 3 years. Three patients had neuropathic DFUs in our study. Whereas in the study by Gardner et al. (2013), out of 52 patients enrolled 43 were male, 48 were white, 43 patients had type 2 diabetes, and nine had type 1 diabetes. All patients had neuropathic DFU with 42 DFUs located on the plantar forefoot and only DFU samples were surveyed (Gardner et al. 2013).

Redel et al. (2013) conducted a case‐control observational study to assess the profile of cutaneous microbiota in arms and feet of men without diabetes and with diabetes but without DFUs using high‐throughput 16S rRNA gene sequencing. Their study, which comprised a mixed‐ethnic population, found that microbiota composition and total bacterial counts were similar in the arm samples of men with and without diabetes. However, plantar foot skin samples contained a greater bacterial diversity with decreased *Staphylococcus* species and increased populations of virulent forms of *S. aureus* in men with diabetes. The relative abundance of *Actinomycetota* was higher in men with diabetes while *Bacillota* was lower (Redel et al. 2013). Our results are consistent with these findings of the detection of these bacteria in patients with diabetes both in DFUs and skin controls at varied levels of relative abundance but we did not study people without diabetes. Since patients without diabetes were not included in our study, a direct comparison in microbiome diversity and composition could not be made between diabetic and nondiabetic skin. Other studies have shown that skin microbiome interacts with the microenvironment and the host in systemic and cutaneous diseases (Grogan et al. 2019; Jo et al. 2017). Consequently, changes in diabetic factors including hyperglycemia and vascular impairment may influence skin microbiota. Indeed, one study that profiled microorganisms obtained from skin swabs from healthy skin, diabetic skin and DFUs using 16S rRNA gene sequencing reported changes in the abundance of *Staphylococcus*, *Corynebacterium*, *Enhydrobacter*, *E. coli*, and *Pseudomonas* in the skin bacterial colonies between these samples and suggested that these changes were the main cause of DFU (Zhang et al. 2023). Therefore, further studies which include skin from patients without diabetes are needed to better understand the microbiome correlation between nondiabetic skin, diabetic skin and DFUs and to explore alterations in microbiome diversity and composition and how these may affect healing of DFUs in the ethnic population enrolled in our study. In another study, Huang et al. (2022) characterized the microbiome of DFUs and intact skin from ulcer swabs and tissue biopsies taken from 10 patients with diabetes using conventional culture and 16S rRNA sequencing. They reported that *Staphylococcus* and *Corynebacterium*, both opportunistic pathogens, were the two most abundant genera found in contralateral intact skin, while *Prevotella* and *Corynebacterium* were the most abundant genera present in DFUs (Huang et al. 2022). In our study, *Corynebacterium, Staphylococcus*, and *Pseudomona*s were the dominant genera found in DFUs while *Corynebacterium, Staphylococcus*, and *Streptococcus* were the abundant genera found in normal skin controls. The differences in bacterial genera observed in our study compared to theirs may be ascribed to the different skin sample locations (DFUs were compared with healthy, undamaged skin adjacent to the wound edge on the same foot in our study compared to contralateral intact skin) and the ethnic groups studied (Afro‐Caribbeans recruited in our study vs. Chinese). In alignment with the latter, Ogai et al. (2022) reported significant differences in the skin microbiome between different racial and geographical groups. The skin microbiome profiles of healthy individuals from Cameroon and Japan were compared and *Micrococcus*, was found to be enriched in Cameroonian skin samples but mostly absent in Japanese skin samples. In contrast, a significantly higher relative abundance of *Cutibacterium* species was found in Japanese compared to Cameroonian skin samples (Ogai et al. 2022). Differences were also reported in both alpha and beta diversity measurements between the two races (Ogai et al. 2022).

A review which surveyed the microbiome of chronic wounds from five microbiome studies utilizing next‐generation sequencing approach, reported that the most bacteria colonizing wounds belonged to 21 families with *Staphylococcaceae* and *Pseudomonadaceae* being the predominant bacteria in chronic wounds including DFUs and venous ulcers (Misic et al. 2014). Similarly, in our study at the family level, *Pseudomonadaceae* showed higher relative abundance in DFUs compared to control skin, whereas *Staphylococcaceae* showed comparable relative abundance between DFUs and controls. In another study which compared the microbiome diversity of DFU swab and tissue samples using 16S rRNA gene sequencing, the presence of the species *Pseudomonas, Staphylococcus, Streptococcus, Corynebacterium, Finegoldia*, and *Anaerococcus*, among others in DFUs was reported (Travis et al. 2020). These bacteria are known as potential pathogens (Travis et al. 2020). Kalan et al. (2019) conducted a longitudinal, prospective study of patients with neuropathic DFUs to investigate the role of colonizing microbiota in diabetic wound healing, clinical outcomes, and response to interventions using metagenomic shotgun sequencing (Kalan et al. 2019). They found *S. aureus, P. aeruginosa, C. striatum*, and *Alcaligenes faecalis*, respectively, as the most abundant species of bacteria detected in all samples. While, in descending order, *Staphylococcus* (18.95%), *Corynebacterium* (14.64%), *Pseudomonas* (9.37%), and *Streptococcus* (7.32%) were the most abundant genera found in DFUs (Kalan et al. 2019). Though the aforementioned studies did not focus on persons of African descent, findings from our study corroborate the presence of these bacteria in our ethnic population. Moreover, the species of *Staphylococcus* genus, were detected in both DFUs and controls with *S. aureus* showing a higher relative abundance in undamaged skin. Interestingly, we detected *Staphylococcus pettenkoferi*, a coagulase‐negative *Staphylococcus*, in DFUs but not in controls. *S. pettenkoferi* has been found to be present in DFUs (Ugaban and She 2022) and a recent study confirmed its pathogenicity (Magnan et al. 2022). Furthermore, studies have shown bacteria such as *P. aeruginosa*, which was detected at a higher mean relative abundance in our DFUs compared to control skin, can be motile (Kazmierczak et al. 2015), which may complicate the use of controls in the same patients.

In our study *Klebsiella, Escherichia, Alcaligenes, Brevibacterium*, and *Anaerococcus* were detected in DFUs but not in control skin confirming other DFU studies (Travis et al. 2020; Makeri et al. 2024; Zhang et al. 2023; Kalan et al. 2019). *Alcaligenes faecalis* typically considered commensal or contaminant has been shown to impact wound severity and healing (Kalan et al. 2019). Hence, our study revealed variation in relative‐abundance profiles and bacterial community composition between DFUs and controls, which may be relevant to DFU healing phenotype. Indeed, other studies have shown that delayed healing observed in chronic wounds is often associated with instability in microbiota composition of the wound and changes in bacterial diversity (Tomic‐Canic et al. 2020). Loesche et al. (2017) evaluated the temporal dynamics of the microbiota colonizing DFUs and reported increased instability of DFU microbiota and rapid and dynamic changes correlated with faster healing and improved outcomes (Loesche et al. 2017).

### 4.2 Fungi

Han et al. (2020) evaluated microbiomes from foot skin swabs from healthy individuals in comparison to those from patients with type 2 diabetes using sequencing of bacterial 16S rRNA gene and the fungal ITS2 region (Han et al. 2020). They found differential skin microbiome, especially for fungi, in patients with diabetes compared to patients without diabetes. Out of 35 biomarkers identified, five were abundant in diabetic samples including *Malasseziomycetes*, *Malasseziales*, *Trichophyton rubrum, Malassezia sp_FR682163*, and *Malassezia restricta*. The authors were unable to further explore the role of the identified components as not all could be assigned at the genera or species level (Han et al. 2020). In contrast, in our study we detected *Rhodotorula* at a higher mean relative abundance in DFUs (6.3%) compared to controls (4.06%), and the two most abundant species detected in our diabetic cohort were *Rhodotorula graminis* (3.5% in controls vs. 6.2% in DFUs) and *P. leucadendri* (3.9% in controls vs. 3.8% in DFUs). *Rhodotorula* is a genus of fungi in the class *Microbotryomycetes* belonging to the division *Basidiomycota*. This common environmental yeast can act as an opportunistic pathogen and can colonize and infect susceptible patients (Wirth and Goldani 2012). Skin swabs were used in the study by Han et al. (2020) whereas tissue biopsies were used in our study. Furthermore, their study was conducted in Korea, in contrast to our study which was conducted in Barbados with persons of African‐Caribbean origin. These factors may have accounted for some of the differences observed between the two studies. In agreement, other studies have reported several factors, including body regions, types of samples obtained, culture versus sequencing techniques, age, sex, ethnicity, host genetics and innate/adaptive immunity, and geographical location, which may cause the differences reported previously in microbiome and mycobiome composition and diversity (Ogai et al. 2022; Costello et al. 2009; Gupta et al. 2017; Wang et al. 2021).

Kalan et al. (2016) conducted a longitudinal study of 100 DFUs under standardized treatment using high‐throughput sequencing of the rRNA ITS1 to explore the dynamic diversity of the mycobiome, its stability in response to host factors, and the association of pathogenic fungi with poor clinical outcomes (Kalan et al. 2016). Seventeen phylotypes were identified all belonging to the phylum *Ascomycota* or *Basidiomycota*. The two most abundant species, both belonging to *Ascomycota* were *C. herbarum* and *C. albicans* (Kalan et al. 2016). The most abundant *Basidiomycota* identified in their study were the *Trichosporon* and *Rhodosporidium* opportunistic yeast pathogens. Similarly, from our study, the resulting OTUs of fungal (5079) communities were classified into three main phyla > 1% relative abundance (predominantly, *Ascomycota*, followed by *Mucoromycota* and *Basidiomycota*), in patients with diabetes in both DFUs and controls.

A high proportion of unclassified fungal sequences was observed across all taxonomic ranks in our study. In particular, the unclassified sequences at species level were recorded as high as 75.6% in DFUs and 82.9% in controls. These findings are consistent with rates reported in other published studies (Nilsson et al. 2019; Wu et al. 2019) and reflects the known incompleteness of current fungal ITS reference databases (Hyde et al. 2024; Phukhamsakda et al. 2022). Notwithstanding, it can also not be ruled out that the high proportion of unclassified fungal sequences at all taxonomic levels found within our study cohort may indicate the presence of novel lineages. The paucity of mycobiome data for Afro‐Caribbean individuals leaves scope for the possibility that this population may harbor distinct fungal communities based on geographical, environmental, dietary, and genetic factors not yet represented in existing reference databases. However, further investigation is warranted using culture‐based methods and whole genome sequencing to improve taxanomic resolution and define the clinical role of these taxa in DFU pathogenesis in this ethnic population.

*Candida* spp. are opportunistic fungal pathogens commonly present on human skin. We found a relatively higher mean relative abundance of *C. duobushaemulonii* (recently reclassified as *Candidozyma duobushaemuli* (Liu et al. 2024) in DFUs (4.4%) compared to controls (2.4%). Other studies have reported its presence in chronic lower extremity wounds and diabetic foot infections (Nobrega de Almeida et al. 2016; Ramos et al. 2018). Therefore, findings from our study cohort highlight the need for future studies to characterize its metabolic functions and virulence potential and explore its clinical implications in the DFU microenvironment.

Studies have shown that most chronic non‐healing wounds are polymicrobial in nature, including the large‐scale retrospective study on a multiracial population conducted by Wolcott et al. (2016) in which 2963 samples of different types of chronic wounds were reported to be polymicrobial (Wolcott et al. 2016). Polymicrobial wound environments contribute to impaired host responses and inhibit wound healing (Pastar et al. 2013). Fungi form part of the skin microbiome albeit their phylogenetic diversity is low compared to their bacterial counterpart (Nguyen and Kalan 2022). Dysbiosis of skin microbiota with increased fungal diversity has been associated with chronic immune‐mediated skin diseases (Schmid et al. 2022; Stehlikova et al. 2019). Further, the presence of fungi in chronic wounds like DFUs can act as scaffold for colonizing bacteria to bind and form biofilms and can ultimately be associated with tissue necrosis and amputation (Kalan et al. 2016; Cheong et al. 2021). Interspecies interactions of *Pseudomonas* with *Candida* (Oates et al. 2012) and *C. albicans* with *Staphylococcus* spp (Kalan et al. 2016; Carolus et al. 2019). have been reported. Furthermore, *C. albicans* and *Citrobacter freundii*, isolated from DFU samples were shown to interact with each other and form mixed‐species biofilms (Kalan et al. 2016) suggesting that fungal‐bacterial interaction may contribute to pathogenesis. Indeed, fungal‐bacterial biofilms in chronic wounds have been shown to enhance microbial survival, increase antimicrobial resistance, exacerbate inflammation, and delay wound healing (Wang et al. 2025). In our study, we detected both fungal and bacterial communities in our cohort, but their interactions were not explored. Further studies are needed to dissect interkingdom interactions and how this may influence DFU and affect the healing process.

### 4.3 DFU Pathogenesis and Clinical Implications

Several taxa detected in the present study have well‐documented functional profiles implicated in DFU pathogenesis. *P. aeruginosa* and *S. aureus* are commonly found in chronic wounds and can generate biofilms, causing delayed wound healing (Tomic‐Canic et al. 2020; Brandenburg et al. 2018; Gurjala et al. 2011; Hirsch et al. 2008). Bacterial biofilms are reported to be involved in more than 78% of all chronic wound infections (Malone et al. 2017). *C. striatum* is also reported to have biofilm‐forming capabilities on both biotic and abiotic surfaces contributing to its persistence in wounds and positive correlation with ulcer duration (MacLeod 2019; Silva‐Santana et al. 2021). Similarly, *Candida* species possess the capacity to form drug‐resistant biofilms and secrete proteolytic enzymes that degrade host tissue (Mayer et al. 2013). Hence, the presence of these microorganisms in our patient samples may affect DFU healing outcome. Indeed, studies have shown that the Ggram‐negative pathogen *P. aeruginosa* is most frequently found in DFUs and its presence in wounds has been linked with poor healing outcomes (Goldufsky et al. 2015). This pathogen has been reported to mediate tissue damage via virulence mechanisms including the type III secretion system, production of tissue‐degrading enzymes, and the release of pyocyanin and rhamnolipids that impair host immune defences and inhibit wound healing (Wang et al. 2025). Moreover, wound communities dominated by *P. aeruginosa* are often linked with an overall reduction in microbial diversity further contributing to delayed wound healing (Al‐Taweel et al. 2025; Tipton et al. 2020). Multi‐drug resistant strains of *P. aeruginosa* are clinically significant because they pose a challenge to wound management (Tipton et al. 2020; Sacks et al. 2018) and because it occupies deeper, oxygen‐depleted wound niches compared to aerobic surface colonizers making it less susceptible to antibiotic treatment (Borriello et al. 2004; Kim et al. 2024). *Staphylococcus*, another known producer of virulence factors, carries antimicrobial resistance determinants that may impair wound healing and complicate clinical management (Malone et al. 2017; Citron et al. 2007). *C. striatum* is an emerging multidrug‐resistant opportunistic pathogen that has been associated with wound infections, bacteraemia, and device‐associated infections, and has been reported to be resistant to a variety of oral antimicrobial agents including penicillin, tetracycline, clindamycin, erythromycin, and ciprofloxacin in up to 71% of isolates tested (Hahn et al. 2016; McMullen et al. 2017). Historically considered a skin commensal, *C. striatum* has significant clinical relevance due to its multidrug‐resistant profile and may contribute to poor clinical outcomes if not specifically targeted in treatment. *C. duobushaemulonii* belongs to the *Candida haemulonii* complex, a group of emerging multidrug‐resistant pathogens with intrinsic resistance to azoles, amphotericin B, 5‐fluorocytosine and the echinocandin anidulafungin (Cendejas‐Bueno et al. 2012; Zhou et al. 2025). Studies have shown that *C. duobushaemulonii* can form biofilms on both biotic and abiotic surfaces (Ramos et al. 2020), a key virulence attribute that augments persistence in wounds and contributes to antifungal resistance. However, more studies are needed to investigate the clinical implications of *C. duobushaemulonii* in the DFU microenvironment. One drawback is the unreliable identification of *C. duobushaemulonii* using conventional phenotypic identification systems with commercial platforms which often misidentify it for *C. haemulonii*, risking incorrect use of antifungal therapy (Cendejas‐Bueno et al. 2012). However, utilizing advanced molecular techniques including MALDI‐TOF mass spectrometry can aid in the reliable identification of *C. duobushaemulonii* (Cendejas‐Bueno et al. 2012; Hou et al. 2016).

## 5 Limitations of the Study

The limitations of this study include the relatively small number of patients and the fact that all participants were male. A small sample size may reflect inter‐individual variability and limit the statistical power to draw robust or generalizable conclusions representative of females and the Afro‐Caribbean population as a whole. The microbiome composition of DFUs in Afro‐Caribbean women may also vary because of hormonal, immunological, and physiological differences. Moreover the small sample size in this pilot study renders statistical adjustment for ulcer duration or systemic clinical variables not possible; hence, these variables were reported but are recognized as potential confounding factors. Future studies including female participants, larger cohorts, and adequate control of confounders are needed.

Another limitation was the close proximity between DFU and intact control skin samples on the same foot as this may have resulted in some shared microbial diversity. However, this was done so that both sites could be sampled from the same local microenvironment, to minimize the confounding effects of differences in anatomical locales and so that each participant served as his own matched control. Furthermore, creating wounds further away from the ulcer sites was deemed inappropriate as this would cause additional trauma to patients who already have difficult‐to‐heal wounds.

Limited representation of fungal species in available reference databases when using ITS‐based sequencing analysis of the fungal communities represents another limitation. Global fungal diversity has been estimated at between 2 and 11 million species, of which only approximately 150,000 have been formally described (Hyde et al. 2024; Phukhamsakda et al. 2022), highlighting the paucity of available sequence data in public reference databases for a large proportion of fungal species. Therefore the high proportion of unclassified sequences is an expected reported outcome of ITS‐based fungal profiling in clinical samples and reflects the known limitations of current fungal ITS reference databases (Hyde et al. 2024; Phukhamsakda et al. 2022).

The current study characterized the taxonomic composition of the DFU microbiome and did not address functional significance such as virulence gene expression, biofilm‐forming capacity, or antimicrobial resistance profiles. However, the taxonomic findings reported provide a foundation for future work incorporating metagenomic or metatranscriptomic approaches to explore both the functional and resistance profiles of the DFU microbiome to help inform treatment choice and antimicrobial stewardship strategies in patients with DFU.

## 6 Conclusions

This pilot study utilized Illumina‐based 16S rRNA gene and ITS2 amplicon sequencing to compare bacterial and fungal taxonomic composition and diversity in tissue biopsies from chronic DFUs and matched intact‐skin controls from Afro‐Caribbean patients with type 2 diabetes in Barbados. Bacterial and fungal communities were diverse in both DFUs and controls, with relative abundances varying between the two groups. The predominant bacterial phyla found were *Pseudomonadota*, *Bacillota*, *Actinomycetota*, and *Bacteroidota*, in agreement with the results of other DFU microbiome studies. At the species level, *C. striatum*, *P. aeruginosa*, and *S. aureus* were some of the predominant bacteria present in DFU wounds and are all associated with clinically important virulence and antimicrobial resistance profiles that could predispose and contribute to wound chronicity and low healing rates. Fungal alpha diversity showed a significantly lower Shannon diversity index in DFUs compared to controls, suggesting that fungal community evenness in the wound environment is reduced. No significant differences were found in bacterial alpha diversity metrics or in beta diversity for either community. *C. duobushaemulonii*, a member of the multidrug‐resistant *C. haemulonii* complex with intrinsic resistance to multiple antifungal agents and shown to form biofilms, was detected with higher mean relative abundance in DFUs than in controls. This may have clinical implications in the DFU microenvironment and therefore warrants further investigation. The high proportion of unclassified fungal sequences across all taxonomic ranks highlights current database limitations but at the same time may represent previously undercharacterised fungal diversity specific to the Afro‐Caribbean microbiome. In this study, the small sample size, and all‐male cohort, was the major limitation of the study. Notwithstanding this study reports the first characterization in Barbados of the bacterial and fungal taxonomic composition of chronic DFUs and matched intact skin in Afro‐Caribbean patients with type 2 diabetes. The observed differences in taxonomic composition and relative abundance profiles, and co‐occurrence of clinically relevant bacterial and fungal taxa, highlight the potential role of polymicrobial communities in DFU chronicity in the Afro‐Caribbean population, and supports future studies to evaluate implications for antimicrobial treatment selection and stewardship.

## Author Contributions

**Nkemcho Ojeh:** conceptualization, formal analysis, writing – review and editing, writing – original draft, resources. **Bidyut R Mohapatra:** conceptualization, formal analysis, writing – review and editing, writing – original draft, resources. **Margaret O'Shea:** conceptualization, writing – review and editing, supervision. **Dale Springer:** conceptualization, writing – review and editing, supervision. **Judy Ward:** methodology, writing – review and editing. **Mohmmed Sallu:** methodology, writing – review and editing. **Natacha Paquette:** methodology, writing – review and editing. **Keith Gooding:** methodology, writing – review and editing. **Anna Springer:** methodology, writing – review and editing. **O. Peter Adams:** conceptualization, writing – review and editing.

## Ethics Statement

The study was conducted in accordance with the Declaration of Helsinki and approved by The University of the West Indies, Barbados Ministry of Health and Wellness Research Ethics Com‐mittee/Institutional Review Board (IRB) (Ref: CREC‐CH.00148/01/2023) and the Queen Elizabeth hospital Research Ethics Committee (Ref: 202021).

## Consent

Informed consent was obtained from all subjects involved in the study.

## Conflicts of Interest

The authors declare no conflicts of interest.

## Supporting information


**Table S1**: Bacterial alpha diversity in DFUs and Controls. **Table S2**: Fungal alpha diversity in DFUs and Controls. **Table S3**: Statistical comparison of prokaryotic alpha diversity indices between DFUs and controls using Bray‐Curtis dissimilarity analysis. **Table S4**: Statistical comparison of fungal alpha diversity indices between DFUs and controls using Bray‐Curtis dissimilarity analysis. **Table S5**: Bacterial beta diversity in DFUs and Controls. **Table S6**: Fungal beta diversity in DFUs and Controls.

## Data Availability

The authors confirm that the data supporting the findings of this study are available within the article. The datasets generated and analyzed during the current study are available in the NCBI BioProject database under the BioProject accession number PRJEB81857 for the 16S rRNA gene sequences and the accession number PRJEB81942 for the ITS region sequences. The data that support the findings of this study are openly available in NCBI BioProject at https://www.ncbi.nlm.nih.gov/bioproject/?term=prjeb81857, reference number PRJEB81857.
